# Carnosic Acid Alleviates BDL-Induced Liver Fibrosis through miR-29b-3p-Mediated Inhibition of the High-Mobility Group Box 1/Toll-Like Receptor 4 Signaling Pathway in Rats

**DOI:** 10.3389/fphar.2017.00976

**Published:** 2018-01-19

**Authors:** Shuai Zhang, Zhecheng Wang, Jie Zhu, Ting Xu, Yan Zhao, Huanyu Zhao, Fan Tang, Zhenlu Li, Junjun Zhou, Dongyan Gao, Xiaofeng Tian, Jihong Yao

**Affiliations:** ^1^Department of Pharmacology, Dalian Medical University, Dalian, China; ^2^Department of General Surgery, Second Affiliated Hospital of Dalian Medical University, Dalian, China

**Keywords:** carnosic acid, bile duct ligation, high-mobility group box-1, miR-29b-3p, liver fibrosis

## Abstract

Fibrosis reflects a progression to liver cancer or cirrhosis of the liver. Recent studies have shown that high-mobility group box-1 (HMGB1) plays a major role in hepatic injury and fibrosis. Carnosic acid (CA), a compound extracted from rosemary, has been reported to alleviate alcoholic and non-alcoholic fatty liver injury. CA can also alleviate renal fibrosis. We hypothesized that CA might exert anti-liver fibrosis properties through an HMGB1-related pathway, and the results of the present study showed that CA treatment significantly protected against hepatic fibrosis in a bile duct ligation (BDL) rat model. CA reduced the liver expression of α-smooth muscle actin (α-SMA) and collagen 1 (Col-1). Importantly, we found that CA ameliorated the increase in HMGB1 and Toll-like receptor 4 (TLR4) caused by BDL, and inhibited NF-κB p65 nuclear translocation in fibrotic livers. *In vitro*, CA inhibited LX2 cell activation by inhibiting HMGB1/TLR4 signaling pathway. Furthermore, miR-29b-3p decreased HMGB1 expression, and a dual-luciferase assay validated these results. Moreover, CA down-regulated HMGB1 and inhibited LX2 cell activation, and these effects were significantly counteracted by antago-miR-29b-3p, indicating that the CA-mediated inhibition of HMGB1 expression might be miR-29b-3p dependent. Collectively, the results demonstrate that a miR-29b-3p/HMGB1/TLR4/NF-κB signaling pathway, which can be modulated by CA, is important in liver fibrosis, and indicate that CA might be a prospective therapeutic drug for liver fibrosis.

## Introduction

Liver fibrosis, a symptom of the progression of chronic liver diseases, is characterized by excessive extracellular matrix (ECM) deposition, distorted hepatic architecture and damaged normal function, which is a main reason for the development of fibrosis into cirrhosis or even hepatocellular carcinoma (Hernandez-Gea and Friedman, 2011; Schuppan and Kim, 2013). In the recent years, the mechanisms underlying the pathogenesis of liver fibrosis have been increasingly studied. Previous studies have reported that the activation of hepatic stellate cells (HSCs) plays an important role in the progression of liver fibrosis (Friedman, 2008; Mallat and Lotersztajn, 2013). HSCs become activated and differentiate into fibroblasts when liver injury occurs; specifically, these cells lose their epithelial characteristics and acquire the characteristics of mesenchymal cells, which show increased expression of α-smooth muscle actin (α-SMA) and Col-1 (Zhou et al., 2007). Therefore, controlling the activation of HSCs is essential to liver fibrosis.

High-mobility group box-1 (HMGB1) as a nuclear non-histone chromosomal protein that can bind to the minor groove of DNA, participates in DNA repair and replication and energy homeostasis (Bianchi and Manfredi, 2007; Kang et al., 2009). Several recent experimental reports have proven that HMGB1 is markedly increased in fibrotic liver diseases (Tu et al., 2012; Seo et al., 2013; Wang et al., 2013; Li X. et al., 2016). HMGB1 is an important inflammatory response mediator (Scaffidi et al., 2002), and HMGB1/toll-like receptors (TLRs) have been shown to play critical roles in liver inflammation and liver fibrosis (Mencin et al., 2009; Berzsenyi et al., 2011; Tu et al., 2012). Following BDL, TLR4-deficient mice exhibits significantly reduced inflammation and hepatic fibrosis, indicating that TLR4 is essential in liver fibrosis (Zhu et al., 2012; Hoshino et al., 2016). Moreover, HMGB1 also promotes the release of pro-inflammatory mediators by acting on its target receptors, leading to nuclear translocation of transcription factors such as NF-κB (Li X. et al., 2016). Therefore, the HMGB1/TLR4/NF-κB signaling pathway plays a pivotal role in liver fibrosis, and modulation of this signaling pathway is an appealing strategy for the inhibition of liver fibrosis.

*Rosmarinus officinalis* L (Lamiaceae) is an herbal plant that is extensively used by the food industry due to its beneficial health properties (Rašković et al., 2014). CA, phenolic compound that is extracted from the leaf of rosemary (Rašković et al., 2014), exhibits many pharmacological activities, including antisteatosis, antioxidant, antiapoptosis and antitumor activities (Jordan et al., 2012; Park and Mun, 2013; Petiwala et al., 2014; Shan et al., 2015). CA can stimulate SIRT1 activity, and subsequently mediates anti-apoptosis by deacetylating downstream factors, including p66Shc and CHREBP (Rašković et al., 2014; Yan et al., 2014; Shan et al., 2015; Gao et al., 2016). CA can alleviate alcoholic and non-alcoholic fatty liver (Rašković et al., 2014; Shan et al., 2015; Gao et al., 2016) and hepatic ischemia reperfusion injury in rats (Yan et al., 2014). Moreover, CA has been reported to alleviate renal fibrosis in rats by mediating the NOX signaling pathway (Jung et al., 2016). However, its effect on hepatic fibrosis and mechanism of action remain unclear. Alcoholic and non-alcoholic fatty liver disease, particularly in the late stages, are more likely to develop into the progression of liver fibrosis. We hypothesized that CA might have anti-liver fibrosis properties.

MicroRNA (miRNAs), which constitute a type of post-transcriptional regulators, are highly conserved, small, non-coding RNAs that bind to the 3′ untranslated region (3′-UTR) complementary sequences of many target mRNAs. miRNAs are important modulators of pathophysiological processes (Neilson and Sharp, 2008; Pirola et al., 2015), are expressed in a developmental stage-specific and tissue-specific manner and specifically reduce mRNA stability (Neilson and Sharp, 2008). In recent years, an increasing number of polyphenols extracted from herbal medicines have been reported to exhibit pharmacological activity by acting on miRNA. Salvianolic acid B inhibits the Hh signaling pathway and reduces liver fibrosis by promoting the expression of miR-152 (Yu et al., 2015). CA exerts antitumor activity by down-regulating miR-15b (Gonzalez-Vallinas et al., 2014), and increases miR-34a to exert an anti-apoptotic effect (Shan et al., 2015).

Based on the miRNA database and our preliminary experiment, we found that miR-29b-3p might play a key role in control of HMGB1 expression in BDL-induced liver fibrosis. The main objectives of this study were as follows: (1) to elucidate the role of the antifibrosis effect of CA in protecting against BDL; (2) to test whether the activity of CA against liver fibrosis is associated with the signaling mechanisms of HMGB1/TLR4/NF-κB pathway in BDL; and (3) to investigate whether CA inhibits HMGB1/TLR4/NF-κB by promoting the expression of miR-29b-3p.

## Materials and Methods

### Reagents

The purity of CA, which was purchased from Shanghai Winherb Medical Science Co., Ltd. (Shanghai, China), was 98%. CA was extracted from rosemary through ethanol extraction, and the coarse extracts were dissolved in macroporous adsorption resin, eluted with different concentrations of alcohol, and concentrated under reduced pressure.

The olive oil used was Aceite De Oliva Virgen Extra of BELLINA and was purchased from Joybuy.com (Beijing, China).

### Experimental Animals

Adult male Sprague-Dawley rats weighing between 180 and 220 g were obtained from the Experimental Animal Center of Dalian Medical University (Dalian, China). CA was dissolved in olive oil. The rats were maintained in a temperature-controlled chamber (22 ± 2°C) with a 12-h light/dark cycle and a relative humidity of 40–60%. The rats had free access to food and water. Forty experimental rats were divided into five groups with eight rats per group: (A) sham-operated; (B) sham+CA (60 mg/kg/day); (C) BDL; (D) BDL-CA (30 mg/kg/day); and (E) BDL-CA (60 mg/kg/day). The abdominal cavities of the rats in Groups A and B were opened, and the common bile duct was isolated but not ligated. The bile ducts of the rats in Groups C, D, and E were ligated (Aubé et al., 2007). The CA concentration administered intragastrically to the rats in Group D was 30 mg/kg/day. The rats in Groups B and E were given CA intragastrically at 60 mg/kg/day, whereas those of Groups A and C were intragastrically given the same amount of solvent olive oil. Twenty-four hours after surgery, the rats in Groups B, D, and E were subjected to the CA treatment for 21 days. After 3 weeks of treatment, the rats were sacrificed by anesthesia after an entire night of fasting. Serum and liver samples were collected for further experiments. The study was subject to approval by the institutional animal care committee of Dalian Medical University. All procedures in this study were performed according to institutional guidelines and the Guide for the Care and Use of Laboratory Animals.

### Cell Culture and Transfection

The human LX2 hepatic cell line was purchased from China Cell Culture Center (Shanghai, China). The cells were cultured in 1640 medium (GIBCO BRL, United States) that containing 10% fetal bovine serum (FBS, GIBCO BRL, United States) in an incubator with 5% CO_2_ at 37°C. Following the manufacturer’s instructions, the cells were treated with 20 μM CA dissolved in DMSO and diluted with DMEM for 12 h before the extraction of total protein or RNA. Transfected experiments were performed using 2 μg pcDNA3.1/HMGB1, 50 nM mimic-miR-29b-3p, 50 nM mimic-miR-300 or 50 nM antagomiR-29b-3p (GenePharma) and Lipofectamine 3000 (Invitrogen, United States) according to the manufacturer’s instructions. pcDNA3.1 (GenePharma) and a random RNA duplex (GenePharma) was used as negative control. After transfection for 24 h, the mimic group was harvested, the pcDNA3.1/HMGB1 and the antagomir group was cultured with or without 20 μM CA for another 12 h. LX2 cells were harvested post-transfection and processed for total RNA and protein extraction. The CA concentration and exposure time were determined based on the previous reports (Shan et al., 2015) combined with the cytotoxicity of CA on LX2 cells and L02 cells (**Supplementary Figure S1**). The cytotoxicty of transfection or plasmid with/without CA on LX2 cells were also assayed (**Supplementary Figure S2**). The supplementary results were obtained by the method described in the part of Supplementary Materials and Methods.

### Serum Levels of Total Bilirubin (Tbil), Alanine Aminotransferase (ALT), and Aspartate Aminotransferase (AST)

Blood samples were collected from the abdominal aorta and centrifuged at 3000 rpm for 15 min to obtain serum. The manufacturer’s recommended protocols (Nanjing Jiancheng Corp, China) were used for measurements of the serum Tbil, ALT and AST levels.

### Liver Histological Examination

Liver tissue samples were embedded in paraffin and cut into 5-μm-thick sections for hematoxylin and eosin (H&E) staining, Masson staining and immunohistochemistry (IHC) staining (Tao et al., 2017). The sections were examined by light microscopy.

### Quantitative RT-PCR

Total RNA from livers and cells was isolated using the TRIzol reagent (TaKaRa, China) according to the manufacturer’s protocols, and reverse transcribed using a TaqMan miRNA Reverse Transcription Kit. The RNA was quantified using an Applied Biosystems 7300 System (Applied Biosystems, United States) using Quantitative RT-PCR (qPCR) with TaqMan miRNA assay Kit (GenePharma Corp, China). The quantity and purity of total RNA samples were tested by UV spectroscopy (Thermo Fisher Scientific, United States). The miRNA expression level was normalized to endogenous expression of RNA U6.

### Western Blotting

Total protein was extracted from liver tissues, and cells were lysed with RIPA buffer, PMSF (Beyotime, China) and Cocktail (Biotool, China). Nuclear extracts were isolated with the Protein Ext Mammalian Nuclear and Cytoplasmic Protein Extraction Kit (TransGen Biotech). The protein from each sample was selected by electrophoresis in 8–12% SDS-PAGE gels (Bio-Rad, United States). These strips were cultured overnight with the designated primary antibodies: HMGB1 (Cell Signaling Technology, United States), TLR4 (Proteintech, China), NF-κB p65 (Proteintech, China), α-SMA (Proteintech, China), Col-1 (Abcam, British), TGF-β1 (ABclonal, China), MMP9 (Wanleibio, China), TGF-β R1 (Wanleibio, China) and GAPDH (Proteintech, China). After incubation with corresponding secondary antibodies at 37°C, bands were exposed and developed after the addition of enhanced chemiluminescence-plus reagents (Advansta, United States). Western blot (WB) images were analyzed with a Gel-Pro Analyzer (Version 5.0; Media Cybernetics, Rockville, MD, United States).

### Dual-Luciferase Reporter

Dual-luciferase reporter plasmids of miR-29b-3p-HMGB1 were purchased from GenePharma Corp. (GenePharma, China). The plasmid and the miR-29b-3p mimic or the miR-29b-3p negative mimic were co-transfected into LX2 cells. Twenty-four hours post-transfection, the firefly and Renilla luciferase activities were measured with a Double-Luciferase Reporter Assay Kit (TransGen Biotec, China), and the firefly luciferase activity was normalized to the Renilla luciferase activity.

### Statistical Analyses

A two-tailed unpaired Student’s t-test or one-way analysis was used for comparison among the groups, and for one-way ANOVA, Tukey’s method was used for statistical analysis. (version 5.0; GraphPad Prism Software, United States). Differences with *P-*value < 0.05 were considered significant.

## Results

### Effects of CA on Liver Injury in BDL Rats

**FIGURE 1:**
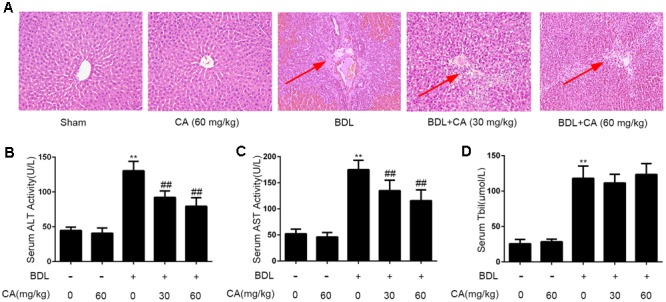
Effects of CA on liver injury in BDL-induced rat models. **(A)** Histological changes in the liver of BDL rats visualized through hematoxylin and eosin (H&E) staining (x100). The hepatic necrosis and foci has been marked with arrows. The experimental groups subjected to H&E staining were as follows: sham; sham + CA (60 mg/kg); BDL; BDL + CA (30 mg/kg); and BDL + CA (60 mg/kg). **(B)** Serum ALT levels (*n* = 8); **(C)** serum aspartate aminotransferase (AST) levels (*n* = 8); **(D)** serum total bilirubin (TBil) levels (*n* = 8). The data are presented as the means ± SD. ^∗∗^*P* < 0.01 versus the sham group; ^##^*P* < 0.01 versus the BDL group.

To confirm the effects of CA on rat liver injury caused by BDL, we performed H&E to determine the degree of liver injury. As indicated by H&E staining, BDL-induced the formation of regenerative nodules and prominent hepatic necrosis in rat liver tissues, and these effects were ameliorated by CA (**Figure 1A**). The dynamic alterations in the liver architecture of BDL rats after 3 weeks of CA treatment were reflected by quantitative biochemical assessments of liver damage. As shown in **Figures 1B–D**), the serum ALT, AST, and Tbil levels clearly increased in response to BDL compared with those of the sham-operated group (*P* < 0.01). CA treatment significantly decreased the ALT and AST levels in a dose-dependent manner (*P* < 0.01). However, the Tbil levels did not change after CA supplementation. These data confirmed that CA protects rats against BDL-induced liver injury.

### CA Ameliorates BDL-Induced Liver Fibrosis in Rats

**FIGURE 2:**
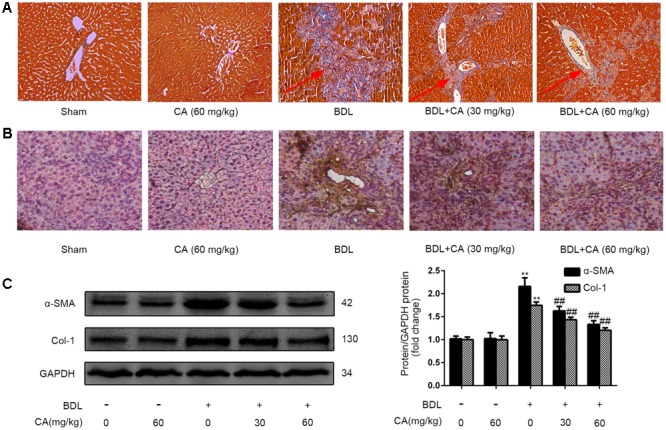
Carnosic acid significantly ameliorates BDL-induced liver fibrosis in rats. **(A)** Masson staining for collagen deposition in rat liver sections (x200). The hyperplasia of the lattice fibers and collagenous fibers has been marked with arrows. **(B)** Immunohistochemistry for α-SMA in rat livers (x200). **(C)** The protein expression of α-SMA and type 1 collagen in the liver was measured by western blot (*n* = 3); the results were normalized relative to the expression of GAPDH. The data are presented as the means ± SD. ^∗∗^*P* < 0.01 versus the sham group; ^##^*P* < 0.01 versus the BDL group.

Hepatic fibrosis is characterized by excessive ECM deposition, particularly type 1 collagens and α-SMA (Hernandez-Gea and Friedman, 2011). The effect of CA against BDL-induced hepatic fibrosis was evaluated through Masson staining, which is a classical histopathological technique used for observing collagen. In BDL rats, extensive accumulation of collagen was observed, and this accumulation was characterized by hyperplasia of the lattice fibers and collagenous fibers in the portal area without ward extension (**Figure 2A**). The expression of α-SMA in liver was revealed by immunohistochemistry staining (**Figure 2B**). It showed that CA supplementation markedly decreased α-SMA expression in the liver. Then we investigated whether CA could ameliorate BDL-induced fibrogenic gene expression by western blot. As is shown, CA significantly reduced the protein expression of α-SMA and Col-1 (**Figure 2C**). These results suggested that CA can alleviate BDL-induced liver fibrosis.

### CA-Mediated Protection against BDL-Induced Liver Fibrosis Involves the Down-Regulation of HMGB1, TLR4, and NF-κB

**FIGURE 3:**
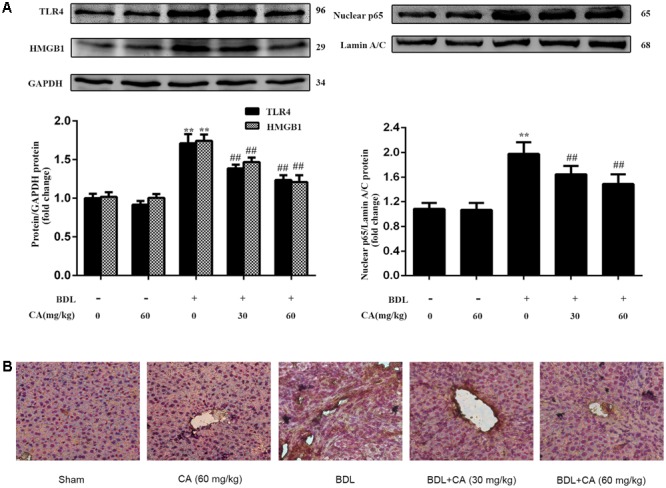
Carnosic acid-mediated protection against BDL-induced liver fibrosis involves the down-regulation of HMGB1, TLR4 and NF-κB. **(A)** The protein levels of HMGB1, TLR4 and nuclear NF-κB p65 in the liver were analyzed by western blot (*n* = 3). **(B)** Immunohistochemistry for HMGB1 in rat livers (x200). The data are presented as the means ± SD. ^∗∗^*P* < 0.01 versus the sham group; ^##^*P* < 0.01 versus the BDL group.

The HMGB1/TLR4/NF-κB signaling pathway induces the proliferation, migration and pro-fibrotic effects of HSCs and enhances the related collagen expression and pro-fibrotic cytokine production in liver fibrosis (Berzsenyi et al., 2011; Zhu et al., 2012; Hoshino et al., 2016). We measured changes in the protein expression of HMGB1 and TLR4 and the nuclear translocation of NF-κB in the liver through western blot. As shown in **Figure 3A**, the HMGB1, TLR4, and nuclear NF-κB p65 expression levels were significantly increased in the BDL group compared with those in the sham-operated group, and this up-regulation was abrogated by CA in a dose-dependent manner. We also observed the expression of the key protein HMGB1 by immunohistochemistry (**Figure 3B**), and the results were consistent with the western blot finding. These findings suggested that CA-mediated protection against BDL-induced liver fibrosis might involve the down-regulation of HMGB1, TLR4 and NF-κB.

### CA Inhibits LX2 Cell Activation by Inhibiting HMGB1/TLR4/NF-κB

**FIGURE 4:**
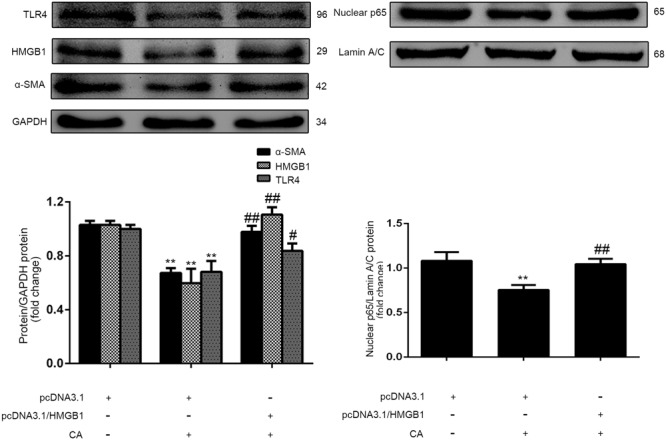
Carnosic acid inhibits LX2 activation by inhibiting HMGB1. LX2 cells were randomly divided into three groups: pcDNA3.1-control, pcDNA3.1-control + CA (20 μM), and pcDNA3.1-HMGB1 + CA (20 μM). After transfection with the plasmid for 24 h, the cells were treated with CA for 12 h, and protein was then extracted. The protein expression levels of α-SMA, HMGB1, TLR4 and nuclear NF-κB p65 in LX2 cells were detected by western blot (*n* = 3). The data are presented as the means ± SD. ^∗∗^*P* < 0.01 versus the control group; ^#^*P* < 0.05 versus the CA group; ^##^*P* < 0.01 versus the CA group.

To determine whether the CA-mediated reduction in the activation of LX2 cells was related to the inhibition of HMGB1 expression, we transfected LX2 cells treated with CA with an HMGB1 over-expression plasmid, and the protein expression levels of α-SMA, HMGB1, TLR4 and NF-κB p65 were detected by western blot. As shown in **Figure 4**, CA decreased the expression of α-SMA, HMGB1, TLR4 and nuclear NF-κB p65 protein in LX2 cells, and this decrease was abrogated by HMGB1 over-expression.

### Regulatory Effect of miR-29b-3p on HMGB1 Expression

**FIGURE 5:**
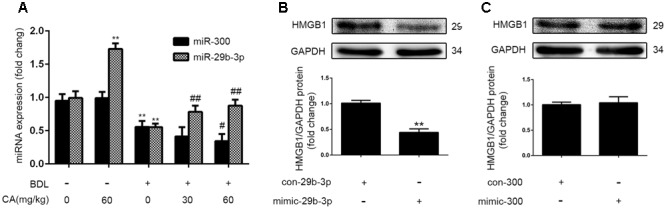
Regulatory effect of miR-29b-3p on HMGB1 expression. **(A)** The levels of miR-300 and miR-29b-3p in the liver of rats were assayed by qPCR (*n* = 6). The data are presented as the means ± SD. ^∗∗^*P* < 0.01 versus the sham group; ^#^*P* < 0.05 versus the BDL group; ^##^*P* < 0.01 versus the BDL group. **(B,C)** LX2 cells were randomly divided into four groups: mimic-miR-29b-3p-control, mimic-miR-29b-3p, mimic-miR-300-control, and mimic-miR-300. The cells were exposed to various treatments for 24 h before extraction of total protein. The HMGB1 protein level in LX2 cells was detected by western blotting (*n* = 3). The data are presented as the means ± SD. ^∗∗^*P* < 0.01 versus the control group.

miRNAs regulate gene expression by binding to the 3′-UTR of target gene mRNAs and control approximately 60% of mammalian genes (Neilson and Sharp, 2008). Therefore, we hypothesized that miRNAs might be associated with the regulation of HMGB1 in BDL-induced liver fibrosis. To investigate the miRNA expression profiles in cholestatic liver, we referred to the literature associated with the BDL model to determine the down-regulated miRNAs (Yang et al., 2012, 2015; Shifeng et al., 2013; Marzioni et al., 2014; Meng et al., 2014; Xiao et al., 2015; Wang et al., 2015) (**Table 1**). Among the 25 down-regulated miRNAs, miR-300 and miR-29b-3p were predicted to bind to the 3′-UTR of HMGB1 mRNA. Therefore, the expression of the two miRNAs was further analyzed by qPCR. The qPCR results indicated that the miR-300 and miR-29b-3p levels were significantly lower (*P* < 0.01) in the BDL rats than in the controls (**Figure 5A**). Based on the miRNA database and the results of our experiment, we determined the effect of mimic-miR-300 and mimic-miR-29b-3p on the expression of HMGB1 in LX2 cells. As shown in **Figures 5B,C**, mimic-miR-29b-3p significantly decreased HMGB1 protein expression, whereas mimic-miR-300 had no effect on HMGB1 protein expression in LX2 cells.

**Table 1:** Down-regulation of putative HMGB1-targeting miRNA in BDL-induced rat model, which through the literature and TargetScan human database (http://www.targetscan.org/vert_70/).

	Down-regulation of putative HMGB1-targeting miRNA in BDL-induced rat model					
Decreased miRNAs	miR-124	miR-138	miR-878	miR-151	miR-543	miR-3593
	miR-23a	miR-10a	miR-295	miR-291a	miR-125b	miR-466c
	miR-300	miR-343	miR-29	miR-329	miR-150	miR-3571
	miR-672	miR-196c	miR-361	miR-802	miR-3068	miR-3068
	miR-3541					
Targetscan	miR-300	miR-29				

### CA Reduces HMGB1 Expression and LX2 Activation by Enhancing miR-29b-3p

**FIGURE 6:**
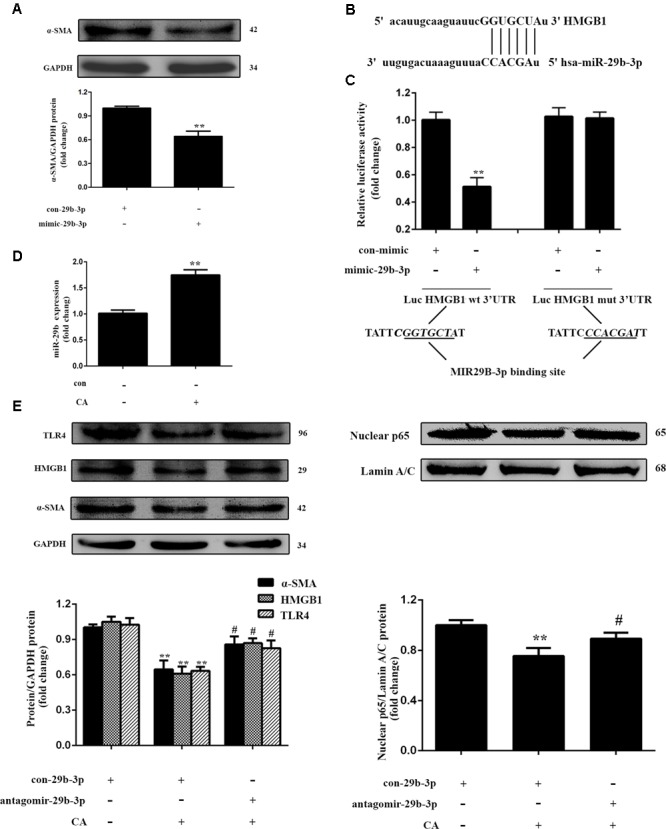
Carnosic acid inhibits HMGB1 expression and LX2 activation by enhancing miR-29b-3p. **(A)** LX2 cells were exposed to mimic-miR-29b-3p for 24 h, and the protein expression of α-SMA in LX2 cells was measured by western blot (*n* = 3). **(B)** Schema representing the functional interaction between miR-29b-3p and the seed sequence in the 3′UTR of HMGB1 as predicted by TargetScanHuman 7.0 (http://www.targetscan.org/vert_70). **(C)** Luciferase assay of LX2 cells co-transfected with reporter constructs containing HMGB1 3’UTRs with (HMGB1-UTRwt) or without (HMGB1-UTRmt) miR-29b-3p-binding sites and the miR-29b-3p mimic or control (*n* = 3). The data are presented as the means ± SD. ^∗∗^*P* < 0.01 versus the control group. **(D)** The level of miR-29b-3p in LX2 cells after CA treatment for 12 h was assayed with qPCR (*n* = 3). The data are presented as the means ± SD. ^∗∗^*P* < 0.01 versus the control group. **(E)** The expression level of α-SMA, HMGB1, TLR4, and nuclear NF-κB p65 protein in LX2 cells were determined by western blot (*n* = 3). For functional analyses, LX2 cells were transfected with antago-miR-29b-3p or antago-miR-29b-3p control. After 24 h, the cells were exposed to 20 μM CA for 12 h. The data are presented as the means ± SD. ^∗∗^*P* < 0.01 versus the control group; ^#^*P* < 0.05 versus the CA group.

To investigate the effects of miR-29b-3p over-expression on the activation of HSCs, α-SMA expression in LX2 cells was determined by western blot. The results revealed that the level of α-SMA was decreased by treatment with mimic-miR-29b-3p (**Figure 6A**), indicating that miR-29b-3p can inhibit the activation of LX2 cells. Given that HMGB1 was predicted to be a putative target of miR-29b-3p (**Figure 6B**), the protein levels of HMGB1 were found to be decreased by mimic-miR-29b-3p (**Figure 5B**). We then generated an HMGB1 3′-UTR luciferase reporter containing the miR-29b-3p-binding sites (HMGB1 wild-type 3′-UTR) or mutated sites (HMGB1 Mut 3′-UTR). The construct was cotransfected into LX2 cells with the miR-29b-3p mimic or the miRNA negative control (con-mimic). The experimental results showed that the miR-29b-3p mimic significantly reduced the luciferase activity driven by the wild-type 3′-UTR of HMGB1 compared with the con-mimic in LX2 cells. Moreover, the luciferase activities of the mutated HMGB1 3′-UTR and the empty vector were not inhibited by the miR-29b-3p mimic (**Figure 6C**). These results confirmed that HMGB1 is a direct target of miR-29b-3p.

For further studies on the effects of CA on HMGB1 in cells, we detected the expression of miR-29b-3p by qPCR. As shown in **Figures 5A, 6D**, the *in vivo*, miR-29b-3p expression was significantly increased by CA treatment, and this increase was positively associated with the protective effect of CA in BDL rats. *In vitro* experiments revealed the same tendency. We subsequently transfected LX2 cells with the miR-29b-3p antagomir in the presence or absence of CA treatment and assessed the expression levels of HMGB1, TLR4, and α-SMA and the translocation of p65 to the nucleus. Significantly lower levels of HMGB1, TLR4, nuclear NF-κB p65 and α-SMA protein were observed in the CA treated compared with the control, and these proteins were increased by the miR-29b-3p antagomir (**Figure 6E**). Therefore, we concluded that CA might target miR-29b-3p to inhibit HMGB1 expression in BDL-induced liver fibrosis. The results showed that CA decreased HMGB1 expression and LX2 activation at least partially in an miR-29b-3p-dependent manner.

### CA-Mediated Protection against BDL-Induced Liver Fibrosis Involves the Down-Regulation of MMP9, TGF-β1, and Its Receptor

**FIGURE 7:**
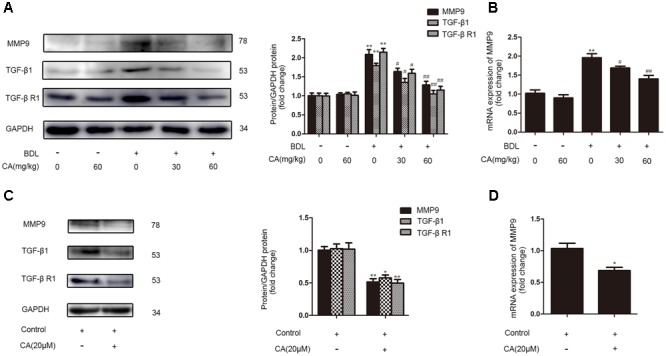
Carnosic acid-mediated protection against BDL-induced liver fibrosis might involve the down-regulation of MMP9, TGF-β1, and its receptor. The expression levels of MMP9 mRNA and MMP9, TGF-β1 and TGF-β R1 protein in livers and LX2 cells were detected. **(A)** The protein levels of MMP9, TGF-β1 and TGF-β R1 in the liver were analyzed by western blot (*n* = 3). **(B)** The mRNA levels of MMP9 in the liver were analyzed by qPCR (*n* = 6). The data are presented as the means ± SD. ^∗∗^*P* < 0.01 versus the sham group; ^#^*P* < 0.05 versus the BDL group; ^##^*P* < 0.01 versus the BDL group. LX2 cells were randomly divided into two groups: control group and CA (20 μM) group. The cells were treated with 20 μM CA for 12 h before the extraction of total protein or RNA. **(C)** The protein levels of MMP9, TGF-β1 and TGF-β R1 in LX2 cells were analyzed by western blot (*n* = 3). **(D)** The mRNA levels of MMP9 in LX2 cells were analyzed by qPCR (*n* = 3). The data are presented as the means ± SD. ^∗∗^*P* < 0.01 versus the control group; ^∗^*P* < 0.05 versus the control group.

Matrix remodeling and the TGF signaling pathway are also important in liver fibrosis (Hu et al., 2009; Li X.M. et al., 2016; Xu et al., 2016; Wu et al., 2017; Yu et al., 2017). We investigated whether CA treatment would alter the expression of MMP9 (a class of enzymes that involved in the degradation of the ECM), as well as TGF-β1 and its receptor, TGF-β R1, in BDL-induced liver fibrosis. The expressions of MMP9, TGF-β1, TGF-β R1 were abnormally increased in the BDL groups, and CA reversed these increases (**Figures 7A,B**). Furthermore, we found the same outcomes in LX2 cells (**Figures 7C,D**), indicating that CA-induced inhibition of hepatic fibrosis might be associated with the down-regulation of MMP9, TGF-β1, and the TGF-β1 receptor.

## Discussion

Carnosic acid, a phenolic compound extracted from *Rosmarinus officinalis* L, is a well-known antiadipogenic and antioxidant agent (Petiwala et al., 2014; Rašković et al., 2014). In recent years, CA has been reported to regulate cell proliferation and differentiation, maintain normal cell function and exhibit antiapoptotic properties (Wang et al., 2011, 2012; Kar et al., 2012). In addition, CA reportedly ameliorates liver injury (Gao et al., 2016) and inflammation (Tang et al., 2016) and inhibits kidney fibrosis by inhibiting the NOX signaling pathway (Jung et al., 2016). However, the mechanisms of hepatic fibrosis regulation by CA are unclear. Here, we generated a BDL-induced liver fibrosis model exhibiting liver fibrosis and injury and we found that CA has favorable characteristics for the treatment of BDL-induced liver fibrosis, as indicated by the improved liver pathology, decreased expression of α-SMA and Col-1. Furthermore, we illuminated the molecular mechanisms through which CA protects against BDL-induced liver fibrosis.

It has been reported that chronic hepatic inflammation could result in hepatic fibrosis and cirrhosis (Schuppan and Kim, 2013). Accumulating evidence indicates that HMGB1 is closely involved in fibrotic diseases, including lung fibrosis, cystic fibrosis, liver fibrosis and pulmonary fibrosis (He et al., 2007; Hamada et al., 2008; Rowe et al., 2008; Tu et al., 2012), whereas the inhibition of HMGB1 signaling can act against experimental models of fibrotic disorders (Ge et al., 2011; Entezari et al., 2012). Moreover, HMGB1 can bind to cell surface receptors, such as RAGE, TLR2, and TLR4, to exert its effects (Mencin et al., 2009; Berzsenyi et al., 2011). In particular, TLR4 plays a crucial role in hepatic inflammation and liver fibrosis (Zhu et al., 2012; Wang et al., 2013; Hoshino et al., 2016). We found that the CA-mediated protection against BDL involves HMGB1/TLR4 down-regulation and the nuclear translocation of NF-κB. Furthermore, CA decreased the expression of α-SMA, HMGB1, and TLR4 protein and nuclear NF-κB p65 in LX2 cells; however, the decreases were abrogated by HMGB1 over-expression. Therefore, CA-induced protection against liver fibrosis is associated with the HMGB1/TLR4/NF-κB pathway.

In recent years, miRNAs have been identified as regulators of many biological processes (Neilson and Sharp, 2008). The silencing and over-expression of miRNAs might be involved in the progression of specific diseases, including fibrosis-related diseases (Sekiya et al., 2011; Yu et al., 2015, 2016). It has been reported that some miRNAs were down-regulated in BDL-induced liver fibrosis (Yang et al., 2012, 2015; Shifeng et al., 2013; Marzioni et al., 2014; Meng et al., 2014; Wang et al., 2015; Xiao et al., 2015). We predicted that miR-300 and miR-29b-3p bind to the 3′-UTR of HMGB1 mRNA and found that the expression of miR-300 and miR-29b-3p in the liver was consistent with that obtained in a previous report. We subsequently discovered that the protein level of HMGB1 decreased only following treatment with the miR-29b-3p mimic. In addition, the expression of α-SMA in LX2 cells was inhibited by mimic-miR-29b-3p. Luciferase assays also confirmed that HMGB1 is a target of miR-29b-3p. These data suggest that miR-29b-3p might play a key role in the control of HMGB1 expression.

The animal experiments revealed that CA treatment could significantly alleviate BDL-induced liver fibrosis in rats and reverse the decrease in miR-29b-3p. Therefore, we hypothesized that CA-induced protection against BDL-induced liver fibrosis occurred through miR-29b-3p up-regulation. We then explored this possibility *in vitro* and found that miR-29b-3p was up-regulated in LX2 cells after CA treatment. In addition, decreased HMGB1 expression was observed in the mimic-miR-29b-3p group compared with that in the control group. Importantly, the miR-29b-3p antagomir could reverse the CA-mediated inhibition of the HMGB1/TLR4/NF-κB pathway and α-SMA levels. Therefore, CA increases miR-29b-3p to inhibit the HMGB1/TLR4/NF-κB pathway, thereby attenuating BDL-induced liver fibrosis.

Effects of drugs on disease is achieved through a variety of channels, while Matrix remodeling and the TGF signaling pathway have essential roles in fibrosis (Hu et al., 2009; Li X.M. et al., 2016; Xu et al., 2016; Wu et al., 2017; Yu et al., 2017). We found that CA provides protection against BDL-induced liver fibrosis by inhibiting MMP9 as well as TGF-β1 and its receptor, TGF-β R1. We will further investigate whether CA inhibits fibrosis relative to matrix remodeling and the TGF signaling pathway. We hope to provide more ideas for future research of CA and liver fibrosis.

In summary, our data indicate that the miR-29b-3p/HMGB1/TLR4/NF-κB signaling pathway might be essential for liver fibrosis and that CA could be a promising therapeutic agent in liver fibrosis by modulating the miR-29b-3p/HMGB1/TLR4 signaling pathway. The antifibrotic functions of CA require further clinical investigation for the treatment of patients with chronic liver diseases.

## Author Contributions

Conceived and designed the experiments: SZ, ZW, ZL, and JY. Performed the experiments: SZ, ZW, JZ, TX, YZ, HZ, and FT. Analyzed the data: SZ, ZW, JjZ, and DG. Wrote and provided suggestion regarding the manuscript: SZ, XT, and JY. Funding support: DG and JY. All authors reviewed the manuscript.

## Conflict of Interest Statement

The authors declare that the research was conducted in the absence of any commercial or financial relationships that could be construed as a potential conflict of interest.

## Supplementary Material

FIGURE S1 Detection of LX2 and L02 cell viability after CA treatment by CCK8 assay. **(A)** Impact of a 12-h treatment with different concentrations of CA, including 0, 5, 10, 20, and 40 μM to the survival rate of LX2 cells. **(B)** Effect of CA (20 μM) on the survival rate of LX2 cells at different time including 0, 3, 6, 12, and 24 h. **(C)** Impact of treatment with CA (20 μM) for 12 h on the survival rate of L02 cells. The data are presented as the means ± SD (*n* = 8). ^∗^*P* < 0.05 versus the control group; ^∗∗^*P* < 0.01 versus the control group.

FIGURE S2 The cytotoxicity of transfection or plasmid with/without CA in LX2 cells was assessed through a CCK8 assay. **(A)** Survival rate of LX2 cells transfected with con-29b-3p/antagomir-29b-3p with/without CA. The cells were divided into five groups: control, con-29b-3p, con-29b-3p + CA, antagomir-29b-3p, and antagomir-29b-3p + CA. **(B)** Survival rate of LX2 cells transfected with pcDNA3.1 or pcDNA3.1/HHMGB1 with/without CA. The cells were divided into five groups; control, pcDNA3.1-control, pcDNA3.1-control + CA (20 μM), pcDNA3.1-HMGB1, and pcDNA3.1-HMGB1 + CA. The data are presented as the means ± SD (*n* = 8). ^∗^*P* < 0.05 versus the control group; ^∗∗^*P* < 0.01 versus the control group; ^#^*P* < 0.05 versus the antagomir-29b-3p or pcDNA3.1-HMGB1 group.

## Abbreviations

α-SMA: α-smooth muscle actin
ALT: alanine aminotransferase
AST: aspartate aminotransferase
BDL: bile duct ligation
CA: carnosic acid
Col-1: collagen 1
HMGB1: high-mobility group box 1
MMP9: matrix metalloprotein 9
NF-κB: nuclear factor kappa B
TGF-β1: transforming growth factor-beta 1
TGF-β R1: transforming growth factor-beta receptor type 1
TLR4: toll-like receptor 4
