# Supplementary figures and images for: Carnosic Acid Alleviates BDL-Induced Liver Fibrosis through miR-29b-3p-Mediated Inhibition of the High-Mobility Group Box 1/Toll-Like Receptor 4 Signaling Pathway in Rats
Source: Front Pharmacol. 2018 Jan 19;8:976. doi: 10.3389/fphar.2017.00976 (PMC5780338; doi:10.3389/fphar.2017.00976)

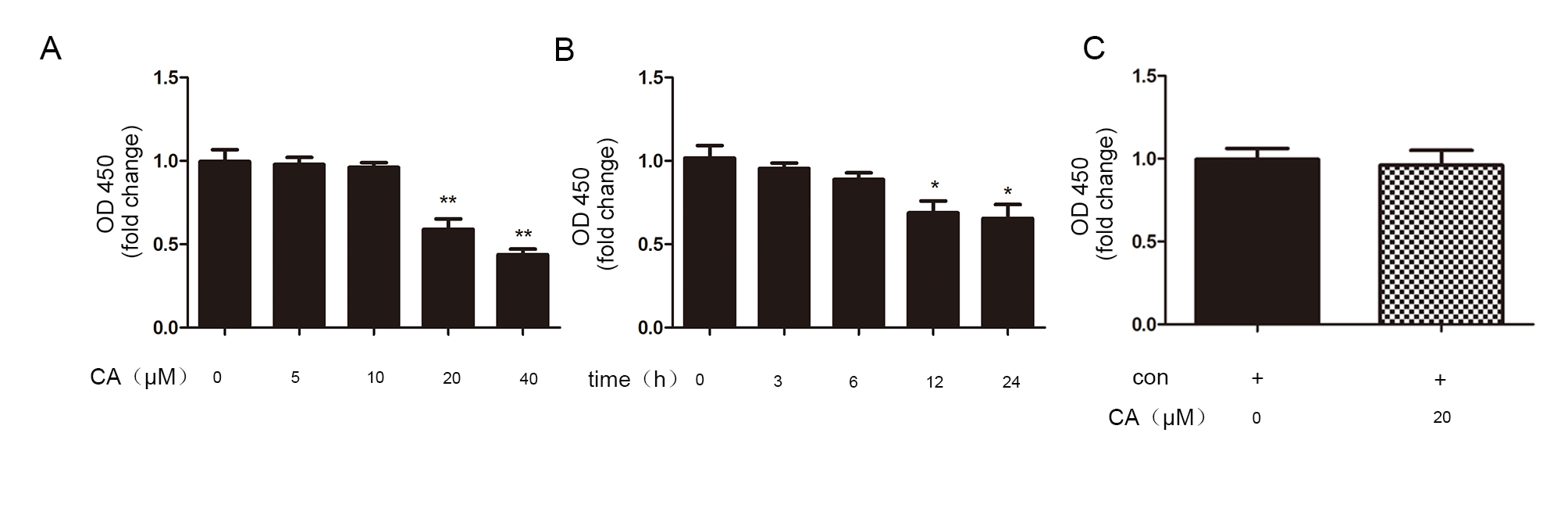

Supplement: FIGURE S1 — Detection of LX2 and L02 cell viability after CA treatment by CCK8 assay. (A) Impact of a 12-h treatment with different concentrations of CA, including 0, 5, 10, 20, and 40 μM to the survival rate of LX2 cells. (B) Effect of CA (20 μM) on the survival rate of LX2 cells at different time including 0, 3, 6, 12, and 24 h. (C) Impact of treatment with CA (20 μM) for 12 h on the survival rate of L02 cells. The data are presented as the means ± SD (n = 8). ∗P < 0.05 versus the control group; ∗∗P < 0.01 versus the control group. [file Image_1.TIF]

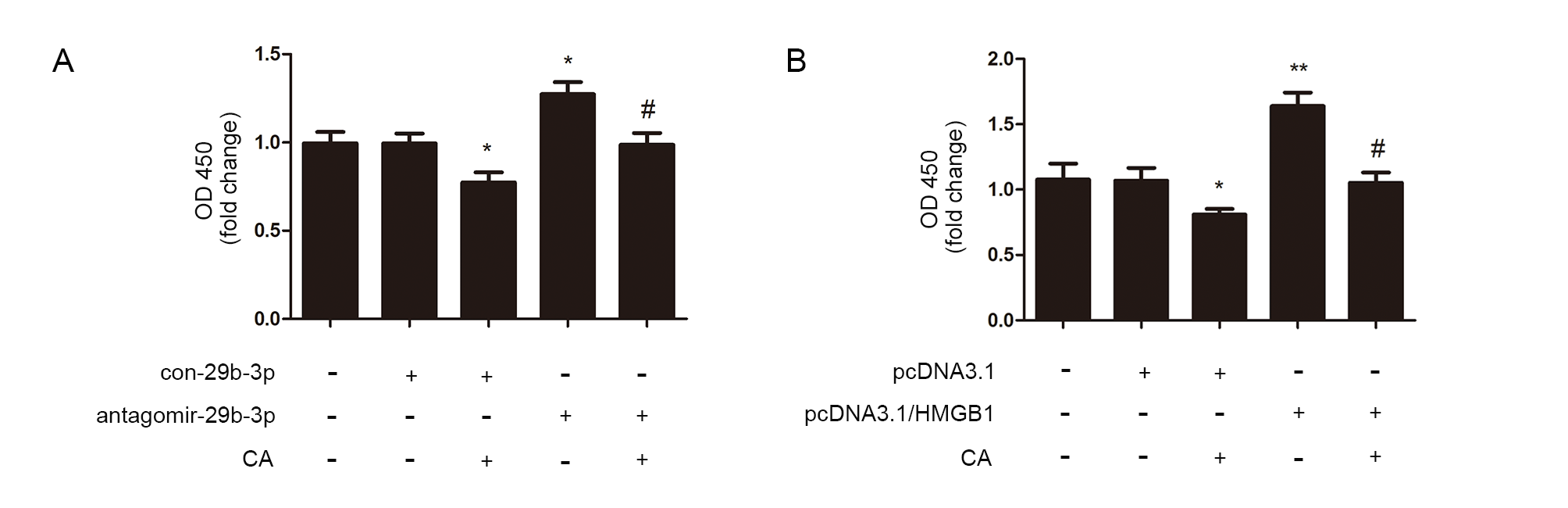

Supplement: FIGURE S2 — The cytotoxicity of transfection or plasmid with/without CA in LX2 cells was assessed through a CCK8 assay. (A) Survival rate of LX2 cells transfected with con-29b-3p/antagomir-29b-3p with/without CA. The cells were divided into five groups: control, con-29b-3p, con-29b-3p + CA, antagomir-29b-3p, and antagomir-29b-3p + CA. (B) Survival rate of LX2 cells transfected with pcDNA3.1 or pcDNA3.1/HHMGB1 with/without CA. The cells were divided into five groups; control, pcDNA3.1-control, pcDNA3.1-control + CA (20 μM), pcDNA3.1-HMGB1, and pcDNA3.1-HMGB1 + CA. The data are presented as the means ± SD (n = 8). ∗P < 0.05 versus the control group; ∗∗P < 0.01 versus the control group; #P < 0.05 versus the antagomir-29b-3p or pcDNA3.1-HMGB1 group. [file Image_2.TIF]
