# Supplementary material for: Carnosic Acid Alleviates BDL-Induced Liver Fibrosis through miR-29b-3p-Mediated Inhibition of the High-Mobility Group Box 1/Toll-Like Receptor 4 Signaling Pathway in Rats
Source: Front Pharmacol. 2018 Jan 19;8:976. doi: 10.3389/fphar.2017.00976 (PMC5780338; doi:10.3389/fphar.2017.00976)
Supplement: Supplementary file 3 [file Presentation_1.PDF]

Supplementary materials and methods

1. Cell culture and transfection

The human LX2 and L02 hepatic cell line was purchased from China Cell Culture Center (Shanghai, China). The cells were cultured in 1640 medium (GIBCO BRL, USA) containing 10% fetal bovine serum (FBS, GIBCO BRL, USA) in an incubator with 5% CO_2 at 37°C. CA (20 μM) was dissolved in DMSO and diluted with DMEM. The LX2 cells were treated 12 h with different concentrations of CA, including 0, 5, 10, 20 and 40μM; the LX2 cells were treated with 20 μM CA for different time including 0, 3, 6, 12 and 24h; the L02 cells were treated with CA (20 μM) for 12 h. Transfected experiments were performed using 2 μg pcDNA3.1/HMGB1 (GenePharma, China) or 50 nM antagomiR-29b-3p (GenePharma) and Lipofectamine 3000 (Invitrogen, USA) according to the manufacturer's instructions. pcDNA3.1 (GenePharma) and a random RNA duplex (GenePharma) was used as negative control. After transfection for 24 h, the pcDNA3.1/HMGB1 and the antagomir group was cultured with or without 20 μM CA for another 12 h.

2. Cell viability assay

Cell viability was measured with a Cell Counting KIT-8 (CCK-8, Biotool, China). The LX2 cells or L02 cells at desired time points, were incubated in CCK-8 solution in a 5% CO_2 incubator at 37°C for 2 h. The intense orange-colored formazan derivative formed by cell metabolism is soluble in the culture medium. The absorbance was measured at 450 nm. Cell number was correlated to optical density (OD).
